# Prosociality and Social Responsibility Were Associated With Intention of COVID-19 Vaccination Among University Students in China

**DOI:** 10.34172/ijhpm.2021.64

**Published:** 2021-06-26

**Authors:** Yanqiu Yu, Sitong Luo, Phoenix Kit-han Mo, Suhua Wang, Junfeng Zhao, Guohua Zhang, Lijuan Li, Liping Li, Joseph Tak-fai Lau

**Affiliations:** ^1^Centre for Health Behaviours Research, JC School of Public Health and Primary Care, The Chinese University of Hong Kong, Hong Kong, China.; ^2^Graduate School of Baotou Medical College, Baotou Medical College, Baotou, China.; ^3^Department of Psychology, School of Education, Henan University, Kaifeng, China.; ^4^Department of Psychology, School of Psychiatry, Wenzhou Medical University, Wenzhou, China.; ^5^School of Public Health, Dali University, Dali, China.; ^6^Shantou University Medical College, Shantou, China.

**Keywords:** COVID-19, Behavioral Intention, Vaccination, Prosociality, Social Responsibility

## Abstract

**Background:** Coronavirus disease 2019 (COVID-19) vaccination is expected to end the pandemic; a high coverage rate is required to meet this end. This study aimed to investigate the prevalence of behavioral intention of free/self-paid COVID-19 vaccination and its associations with prosociality and social responsibility among university students in China.

**Methods:** An anonymous online cross-sectional survey was conducted among 6922 university students in five provinces in China during November 1-28, 2020. With informed consent, participants filled out an online survey link distributed to them via WeChat study groups. The response rate was 72.3%.

**Results:** The prevalence of behavioral intentions of free COVID-19 vaccination was 78.1%, but it dropped to 57.7% if the COVID-19 vaccination involved self-payment (400 RMB; around 42 USD). After adjusting for background factors, prosociality (free vaccination: adjusted odds ratio [ORa] = 1.10, 95% CI: 1.09-1.12; self-paid vaccination: ORa = 1.08, 95% CI: 1.07-1.09) and social responsibility (free vaccination: ORa = 1.17, 95% CI: 1.14-1.19; self-paid vaccination: ORa = 1.13, 95% CI: 1.11-1.14) were positively associated with the two variables of COVID-19 vaccination intention.

**Conclusion:** The present study demonstrated the positive effects of prosociality and social responsibility on the intention of COVID-19 vaccination. Accordingly, modification of prosociality and social responsibility can potentially improve COVID-19 vaccination. Future longitudinal and intervention studies are warranted to confirm such associations across populations and countries.

## Background

**Key Messages**

**Implications for policy makers**

- The prevalence of behavioral intention of free coronavirus disease 2019 (COVID-19) vaccination was close to 80%, yet it dropped down to below 60% if COVID-19 vaccination involved self-payment among university students in China.
- Prosociality and social responsibility were determinants of intention of COVID-19 vaccination.
- Modification of prosociality and social responsibility is potentially possible for promoting COVID-19 vaccination, though it requires future evaluation.

**Implications for the public**

Although the number of daily new coronavirus disease 2019 (COVID-19) cases was low or close to zero in China, herd immunity (against COVID-19) via vaccination is still required to ultimately end the pandemic. Among university students in China, the prevalence of intention of free COVID-19 vaccination was relatively high (close to 80%); it dropped to below 60% if the COVID-19 vaccination involved self-payment. Besides self-protection, COVID-19 vaccination could protect others who are in close contacts and even the society at large (via herd immunity). This study found that prosociality and social responsibility were significantly and positively associated with the intention of COVID-19 vaccination, suggesting that promotion of prosociality and social responsibility is potentially effective to improve COVID-19 vaccination.

Coronavirus disease 2019 (COVID-19) vaccination is certainly the most promising preventive measure to terminate the extremely devastating pandemic. A high population coverage rate of >75% would be required to achieve herd immunity against COVID-19, even if the vaccine was 80% effective.^1^ However, there is a strong global concern about COVID-19 vaccination hesitancy.^2^ The prevalence of intention of COVID-19 vaccination in the general population varied substantially across regions (38.0%-93.3%).^3-7^ Such hesitations may be attributed to the prime concern about safety, which might have been amplified by the wide-spread lack of trust and anti-vaccine attitudes.^8,9^ It is warranted to understand the factors of COVID-19 vaccination. In literature, such factors were mostly confined to socio-demographics (eg, age and gender), cognitions (eg, perceived risk and perceived efficacy of COVID-19 vaccines), and trust toward science and COVID-19-related information.^3-5^

 Perceived benefit (outcome expectancy) is a strong determinant of vaccination behaviors/intentions,^3,10^ as prescribed by a number of behavioral health theories, such as the Health Belief Model^11^ and the Health Action Process Approach.^12^ While perceived benefit of health-related behaviors is often egoistic and “self-directed,” it could also be “others-directed.” For instance, a previous study showed that perceived benefits of both self-protection and protecting others were associated with taking up antiretroviral treatment among Chinese men who have sex with men having high cluster of differentiation 4 (CD4) levels.^13^ Altruism is especially important for the adoption of preventive behaviors against infectious diseases.^14-16^ For instance, the use of face mask among those with flu symptoms to prevent spreading of the virus to others might involve little egoistic benefits but altruistic intention instead.^14^ A study found that self-interest (outcome-for-self) and altruism (outcome-for-others) were both associated with intention of influenza vaccination, although self-interest showed a stronger association than altruism; the modeling exercise further showed that the increase in altruism would reduce the total influenza-related cost, morbidity, and mortality in the community.^15^ It is hence essential to understand factors of COVID-19 vaccination from a perspective considering benefits to others and responsibility to society at large.

 Prosociality is defined as individuals’ enduring tendencies to enact behaviors such as sharing, helping, caring, and empathy.^17^ Prosociality is especially relevant to COVID-19 vaccination. While the perceived benefit of self-protection provides a strong motivation and is a significant factor of COVID-19 vaccination intention,^16^ COVID-19 vaccination can clearly protect others (including both significant others and strangers) by protecting oneself first. Furthermore, the tendency to protect others is of particular importance as COVID-19 is highly infectious, and single super-spreaders could transmit the virus to large clusters of people,^18^ and may lead to serious consequences (eg, deaths), especially among older people and those with chronic diseases.^19^ Prosocial messages have been used effectively to promote vaccination decision such as influenza vaccination and HPV vaccination.^20-22^ Also, several experimental studies found that prosociality was positively associated with preventive behaviors against COVID-19 (eg, social distancing and facemask wearing).^23,24^ However, there is a dearth of studies looking at the relationship between prosociality and COVID-19 vaccination intention. Similar to influenza vaccination, an experiment study found that exposure to messages about perceived benefits of both self-protection and protecting others as a prosocial behavior would increase willingness to take up COVID-19 vaccination, although perceived benefits of self-protection showing a larger effect size.^16^ To our knowledge, no study has looked at the association between the two in non-experimental settings.

 In addition to prosociality, COVID-19 vaccination involves social responsibility, which refers to an individual’s obligation to work and cooperate with others for the benefit of society at large.^25^ The ultimate goal of COVID-19 vaccination programs is to protect the entire population. Thus, besides COVID-19 vaccination’s direct protection for others, the indirect effect of protecting everyone through achievement of herd immunity may be even more important, as it can potentially avoid many infections and deaths. Mass COVID-19 vaccination is required to achieve herd immunity.^1^ The other end of the social responsibility spectrum is the free-riding behavior that some people who could take up COVID-19 vaccination but opt out to save costs (eg, money, time, or side effects) because most or many people around him/her had taken up the vaccination.^22^ In this sense, COVID-19 vaccination is a collective behavior, under which everyone’s contribution is important and required to achieve the ends. Social responsibility is hence involved as everyone has an obligation to contribute to herd immunity. To our knowledge, no study has looked at the relationship between social responsibility and COVID-19 vaccination intention.

 Given the background, this study investigated the prevalence of behavioral intention of free and self-paid COVID-19 vaccination and their associations with prosociality and social responsibility among university students in mainland China. As COVID-19 vaccination has just been approved for use in a number of countries and had not been approved and rolled out at the time of the survey, this study looked at behavioral intention of COVID-19 vaccination. Behavioral intention is one of the strongest predictors of actual behaviors.^26^

## Methods

###  Data Collection

 An anonymous cross-sectional survey was conducted during November 1–28, 2020 among university students in five provinces (Zhejiang, east China; Yunnan, southeast; Guangdong, south; Inner Mongolia, north; and Henan, central) of China via an online survey link. A total of 165 classes of various grades (eg, year 1 to 4) within the faculties of arts, sciences, social sciences, economics or management, engineering, and medicine or pharmacy (and others) of the participating universities were selected by convenience sampling. The collaborating teachers and student helpers sent an invitation message, the online survey link, and several reminders to all the students in the selected classes via WeChat groups that were being used for class administration. The inclusion criteria of participants included being a full-time student at the selected universities and able to read and write Chinese. The questionnaire was self-administered and took about 10-15 minutes to complete. It was written on the invitation message and the online questionnaire that the participation was anonymous, voluntary, and confidential, and that the return of the completed questionnaire implied informed consent. Upon completion, the participants could join a lottery draw which offered eight prizes of 10-50 RMB (about 1.5-7.5 USD) and a symbolic “lucky money” of 1 RMB for half of the participants in each participating university. A published paper that investigated depression among quarantined university students during the COVID-19 outbreak in China used the same methodology.^27^ A total of 9593 invitations had been sent out; 6940 students returned the completed questionnaires (response rate of 72.3%), 18 of which were excluded by quality control; 6922 participants were included in the final data analysis.

###  Measures

####  Background Information

 Background information was collected, including studied province, gender, ethnicity, faculty, grade, and perceived risk (“If not taking up COVID-19 vaccination, what is the chance that you would contract COVID-19 in the future one year;” 1 = extremely low to 5 = extremely high).

####  Behavioral Intention of COVID-19 Vaccination

 Two items assessed participants’ perceived chance of taking up free or self-paid (400 RMB; about 62 USD) COVID-19 vaccination of 80% effectiveness and rare mild side effects within the first six months upon the vaccines’ availability (1 = definitely not to 5 = definitely yes).

####  Prosociality

 Prosociality was assessed by using the 4-item Prosocial Behavioral Intention Scale, which has been validated and showed satisfactory psychometric properties among university students.^28^ The scale was translated into Chinese and back-translated into English by two bilingual researchers. The Chinese version was then finalized by another independent bilingual researcher who is experienced in public health, psychology, and behavioral science. A sample item was “Comfort someone I know after they experience a hardship.” The items were rated with seven-point Likert scale (1 = definitely would not do this to 7 = definitely would do this); higher scores indicated higher levels of prosociality. The Cronbach’s alpha was 0.90 in this study.

####  Social Responsibility

 Social responsibility was assessed by using the 7-item Social Responsibility Scale, which has been applied in adults and showed satisfactory psychometric properties.^25^ Again, the scale was translated into Chinese by two independent bilingual researchers, which was then finalized by a third experienced bilingual researcher. A sample item is “Every person should give some of his time for the good of his town or country.” The items were measured by using five-point Likert scales (1 = strongly disagree to 5 = strongly agree); higher scores indicated higher levels of social responsibility. The Cronbach alpha was 0.68 in this study.

###  Statistical Analysis

 The two outcomes of behavioral intention of free and self-paid COVID-19 vaccination were recoded into binary dependent variables in this study (Likely/definitely yes versus Else). The dichotomization has been done in many previous vaccination behavior research,^29-31^ including those related to COVID-19 vaccination.^3,8,9^ It hence allows for comparisons and facilitates the estimation of the number of people vaccinating. Pearson or Spearman correlation coefficients were derived to examine the correlations among prosociality, social responsibility, and the two intention outcomes. Univariable logistic regression analysis was conducted to test the associations between background factors and the two intention outcomes. Adjusted odds ratios (ORa) were derived for the associations between the independent variables (prosociality and social responsibility) and the dependent variables of vaccination intention, after adjustment of background factors. The analyses were conducted by using SPSS 21.0. Statistical significance was defined as two-tailed *P*< .05.

## Results

###  Descriptive Statistics

 Close to or more than half of the participants were females (63.6%), first-year students (43.2%), and majored in medicine (50.9%). The majority were Han ethnic (86.8%). The mean (SD; range) of the scales were: perceived risk: 2.5 (0.9; 1-5); prosociality: 20.9 (4.6; 4-28); social responsibility: 25.0 (3.5; 12-35) (see Table 1). The prevalence of behavioral intention of free and self-paid (400 RMB) COVID-19 vaccination of 80% effectiveness and rare mild side effects within the first six months upon vaccines’ availability was 78.1% and 57.7%, respectively (see Figure).

**Table 1:** Descriptive Statistics

	**n/Mean**	**%/SD**
**Background Factors**		
Studied province		
Inner Mongolia	2597	37.5
Henan	1943	28.1
Zhejiang	931	13.4
Yunnan	896	12.9
Guangdong	555	8.0
Gender		
Female	4402	63.6
Male	2520	36.4
Ethnicity		
Else	913	13.2
Han	6009	86.8
Faculty		
Art; Social science; Economics and management	1637	23.6
Science; Engineering	1522	22.0
Medicine	3525	50.9
Others	238	3.4
Grade		
First year	2993	43.2
Second year	1894	27.4
Third year	1164	16.8
Fourth/fifth year	776	11.2
Postgraduate	95	1.4
Perceived risk (range = 1 to 5)	2.5	0.9
**Studied variables **		
Prosociality (range = 4 to 28)	20.9	4.6
Social responsibility (range = 12 to 35)	25.0	3.5

Abbreviation: SD, standard deviation.

**Figure:**
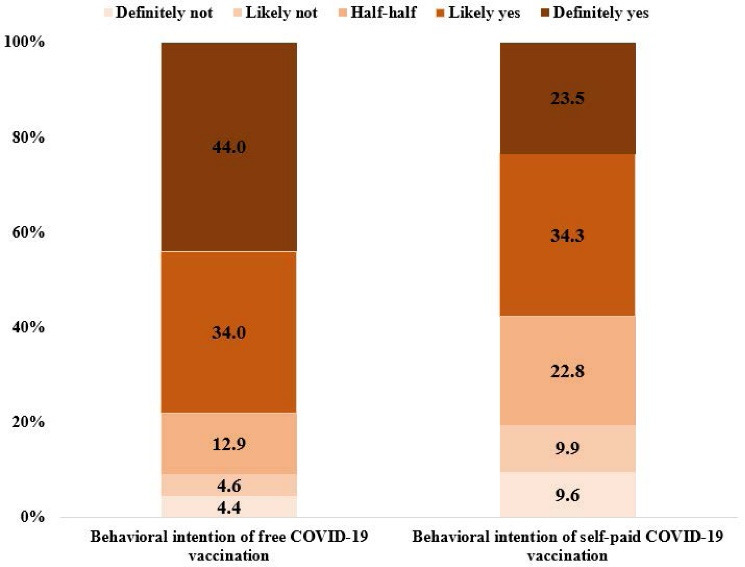
Proportions of behavioral intention of free/self-paid COVID-19 vaccination. Abbreviation: COVID-19, Coronavirus disease 2019.

###  Factors of Behavioral Intention of Free or Self-paid COVID-19 Vaccination Tested by Logistic Regression Analysis

 The location of the university (province), gender, and year of study were significantly associated with the intention of free COVID-19 vaccination, while higher perceived risk, province, year of study were associated with the intention of self-paid COVID-19 vaccination (see Table 2). Adjusted for these background variables, prosociality and social responsibility were both significantly and positively associated with the two variables of vaccination intention (see Table 3); the logit fitting plots of these relationships are presented in supplementary materials (Supplementary file 1, Figure S1).

**Table 2:** Background Factors of Intention of COVID-19 Vaccination (Univariable Logistic Regression Analysis)

	**Behavioral Intention of Free COVID-19 Vaccination**	**Behavioral Intention of Self-paid COVID-19 Vaccination**
	**Likely/Definitely Yes**	**Likely/Definitely Yes**
	**ORc (95% CI)**	**ORc (95% CI)**
Studied province		
Inner Mongolia	Ref = 1.0	Ref = 1.0
Henan	1.44 (1.24-1.66)^a^	0.86 (0.76-0.97)^c^
Zhejiang	1.03 (0.86-1.22)	0.85 (0.73-0.99)^c^
Yunnan	1.55 (1.28-1.89)^a^	1.05 (0.90-1.23)
Guangdong	1.36 (1.08-1.70)^b^	0.84 (0.70-1.01)
Gender		
Female	Ref = 1.0	Ref = 1.0
Male	0.86 (0.77-0.97)^c^	0.92 (0.84-1.02)
Ethnicity		
Else	Ref = 1.0	Ref = 1.0
Han	1.17 (0.99-1.38)	0.92 (0.80-1.06)
Faculty		
Art; Social science; Economics and management	Ref = 1.0	Ref = 1.0
Science; Engineering	0.92 (0.77-1.09)	0.87 (0.75-1.00)
Medicine	0.88 (0.76-1.02)	0.97 (0.87-1.10)
Others	0.80 (0.58-1.10)	0.98 (0.75-1.30)
Grade		
First year	Ref = 1.0	Ref = 1.0
Second year	0.81 (0.70-0.93)^b^	0.72 (0.64-0.81)^a^
Third year	1.00 (0.84-1.18)	0.79 (0.69-0.91)^b^
Fourth/fifth year	0.78 (0.65-0.94)^b^	0.75 (0.64-0.88)^a^
Postgraduate	0.96 (0.58-1.59)	0.89 (0.59-1.35)
Perceived risk	1.00 (0.94-1.07)	1.09 (1.04-1.15)^b^

Abbreviations: ORc, crude odds ratios; Ref, reference group; COVID-19, coronavirus disease 2019. ^a^ *P *< .001; ^b^*P *< .01; ^c^*P *< .05.

**Table 3:** Psychosocial Factors of Intention of COVID-19 Vaccination (Logistic Regression Analysis Adjusted for Background Variables)

	**Behavioral Intention of Free COVID-19 Vaccination**	**Behavioral Intention of Self-paid COVID-19 Vaccination**
	**Likely/Definitely Yes**	**Likely/Definitely Yes**
	**ORc (95% CI)**	**ORc (95% CI)**
Prosociality	1.10 (1.09-1.12)^a^	1.08 (1.07-1.09)^a^
Social responsibility	1.17 (1.14-1.19)^a^	1.13 (1.11-1.14)^a^

Abbreviations: ORc, crude odds ratios; COVID-19, coronavirus disease 2019. ^a^ *P *< .001. Note: The models were adjusted for background factors, including studied province, gender, ethnicity, faculty, grade, and perceived risk.

###  Correlation Analysis

 The two independent variables (prosociality and social responsibility) were both significantly and positively correlated with the intentions of free or self-paid COVID-19 vaccination (*r* = 0.17 and 0.18, respectively; *P*< .001). The two independent variables were significantly correlated with each other (*r* = 0.44; *P*< .001). The intention of free vaccination was also positively correlated with intention of self-paid vaccination (*r* = 0.53; *P*< .001) (see Table 4).

**Table 4:** Correlations Among the Main Studied Variables

	**1**	**2**	**3**	**4**
1. Prosociality	-			
2. Social responsibility	0.44^a^	-		
3. Behavioral intention of free COVID-19 vaccination	0.18^a^	0.21^a^	-	
4. Behavioral intention of self-paid COVID-19 vaccination	0.17^a^	0.18^a^	0.53^a^	-

Abbreviation: COVID-19, coronavirus disease 2019. ^a^ *P *< .001. Note: Pearson correlation coefficient was derived for the correlation between prosociality and social responsibility, while the rest were Spearman correlation coefficients.

## Discussion

 University students form an important subgroup of COVID-19 vaccination due to their highly concentrated living/learning spaces and frequent daily interactions, which may make social distancing less feasible. The observed prevalence of intention of free COVID-19 vaccination (about 80%) was relatively high but was lower than that of the university students in Italy (86.1%)^32^ and the Philippines (81.3%),^33^ and that of Chinese adults in general (91.3%).^5^ It is noteworthy that the prevalence of intention of COVID-19 vaccination dropped to <60% if the vaccination involved self-payment (400 RMB; about 62 USD); the Chinese government needs to provide free vaccination to university students. The actual prevalence of COVID-19 vaccination among university students might be substantially lower. First, literature shows that many of those with a behavioral intention would not actually act subsequently.^34^ Second, the high prevalence of 80% was based on the scenario of rare mild side effects but both mild and severe adverse effects of COVID-19 vaccination have been commonly reported.^35^ Hence, there are substantial rooms and needs for promoting COVID-19 vaccination among university students in China.

 The findings show that university students possessed a relatively high level of prosociality, as seen by the high mean score of 20.9 (range = 4-28). COVID-19 vaccination can be regarded as a prosocial behavior as it certainly protects others. Supporting the alternate hypothesis, the present study found a significant positive association between prosociality and COVID-19 vaccination intention. Prosocial behaviors frequently occurred and have played important roles during public health crisis and disasters (eg, the Wenchuan earthquake in 2008 in China,^36^ the Hurricane Katrina in the United States,^37^ and the 911 terrorist attack^38^). In a previous study, over 60% of the Italian adults self-reported having performed some prosocial behaviors (eg, donation of money/medical supplies, sharing online verified health information with others, and helping a neighbor) during the COVID-19 pandemic, which were associated with perceived community resilience.^39^ The finding of the present study has hence extended our understanding about the potential importance of prosociality in controlling the present and future pandemics.

 The two constructs of prosociality and social responsibility are, however, distinctive, as the correlation between these two variables only explained about ¼ of the variance. The level of social responsibility was also relatively high; the mean score was 25.0 (range = 12-35). In the present study, social responsibility was also significantly associated with the intention of COVID-19 vaccination. Although some studies have looked at the role of prosociality in affecting influenza vaccination and COVID-19 vaccination intention, to our knowledge, no study has looked at COVID-19 vaccination intention from the angle of social responsibility. Social responsibility is an element of social capital^40^; the latter can be “understood roughly as the goodwill that is engendered by the fabric of social relations and that can be mobilized to facilitate actions” (p. 17), which is commonly known as a determinant of health behaviors.^41^ In this case, social responsibility makes citizens to put aside their self-interest and increases the ability to act collectively to deal with shared challenges and crisis faced by the community, such as taking up COVID-19 vaccination to achieve herd immunity against COVID-19.

 Prosociality and social responsibility are universal attributes. It is contended that their significant positive associations with the intention of COVID-19 vaccination would be found in other countries. Such findings might, however, be mixed, as a previous experimental study found a non-significant association between prosociality and the intention of COVID-19 vaccination.^42^ The level and the strength of the associations between prosociality/social responsibility and COVID-19 vaccination intention are likely to vary across socio-cultural contexts. A global health perspective is warranted. Generalization would allow researchers, health workers, and policy-makers to understand better the global community responses to the COVID-19 pandemic and take actions to increase COVID-19 vaccination rates of different countries.

 The findings point to some new research directions to understand the roles of prosociality and social responsibility in controlling COVID-19 and other pandemics. Regarding COVID-19 vaccination, it is inevitable that some people are ambivalent about their prosociality to benefiting and protecting others (others’ interest) versus the safety concern and/or time/cost required for vaccination (self-interest). A few specific and potentially important research questions emerge. First, future studies may investigate the relative strength of the associations between potentially conflicting self-directed versus others-directed interests (eg, prosociality versus safety concern) and COVID-19 vaccination intention and behavior. Second, it is possible that prosociality/social responsibility may moderate the associations between COVID-19- related cognitive factors (eg, perceived risk of contracting COVID-19) and COVID-19 vaccination behavior/intention. For instance, perceived risk might become less important in the presence of strong prosociality in affecting COVID-19 vaccination decision. Third, both levels of COVID-19-related preventive behaviors and the number of infections may vary dramatically across countries. An important policy research question is whether and how much such inter-country variations in the number of COVID-19 infections would be explained (mediated) by the variations in prosociality and social responsibility across countries. Fourth, it is interesting to understand the inter-relationship between culture (eg, collectivism versus individualism), prosociality/social responsibility, and COVID-19 vaccination. Future studies are warranted to answer these relatively new research questions.

 Prosociality can be enhanced through interventions, such as those involving community services and moral elevation.^43^ Better explanations about requirement/social benefits related to herd immunity and vaccination coverage and establishment of social norms may also foster public awareness and social responsibility related to COVID-19 vaccination. Although prosocial messages were effective in improving a number of vaccination behaviors (eg, influenza vaccination) and behavioral intention of COVID-19 vaccination in experimental studies,^16,20-22^ readers are cautioned that the present study only identified associations instead of causal relationships, nor intervention efficacy; it does not come to the conclusion about effectiveness of the afore-mentioned interventions. Future studies are needed to evaluate the efficacy of health promotion of COVID-19 vaccination via enhancement of prosociality and social responsibility. In addition, meta-analyses have shown high efficacy of motivational interviewing in changing behaviors by evoking ambivalence about the behavior and facilitating individuals in understanding his/her own situations and set goals for behavioral change.^44^ This method has been recommended by the WHO and the United States Centers for Disease Control and Prevention (CDC) to improve COVID-19 vaccination uptake rates^45^ and seems to fit the context of potential mental conflicts about safety concerns and others-directed interest (ie, prosociality and social responsibility).

 This study has several limitations. First, as afore-mentioned, causal and temporal inferences cannot be made due to the cross-sectional nature of the survey. Second, as COVID-19 vaccination and showing prosociality/social responsibility are socially desirable, social desirability biases may exist, which may inflate the levels of these three variables. Third, although the five studied provinces cover different regions of China and had a relatively high response rate (>70%), generalization of the results to other Chinese cities/regions should be made cautiously. Fourth, the amount of monetary cost for self-paid COVID-19 vaccination was set arbitrarily. Last, concerning the question about the intention of self-paid vaccination, we did not specify whether the participants expected free COVID-19 vaccines would be available to them concomitantly with self-paid vaccines. It is possible that some of them did not intend to take up self-paid vaccines as they believe that they would be given free vaccines even if they did not pay for such. Thus, the lack of intention to take up self-paid vaccination does not mean that these students would not get vaccinated. Nevertheless, the finding depicted what might happen if only self-paid vaccines would be available to university students.

 In conclusion, the prevalence of behavioral intention of COVID-19 vaccination among Chinese university students was relatively high. Yet, health promotion is still needed given high frequencies of side effects. It is innovative to look at the role of people’s prosociality and social responsibility traits on COVID-19 vaccination in Chinese societies. Future longitudinal and intervention studies are warranted to confirm the findings across cultures and populations. Randomized controlled trials to evaluate efficacy of intervention in improving COVID-19 vaccination rate via enhancement of prosociality and social responsibility are also warranted.

## Acknowledgement

 We would like to thank Wenjie Hou from the Baotou Medical College, Dongdong Gao from the Henan University, Xiaolian Tu, Nani Ding, Jingjing Zhang from the Wenzhou Medical University, and Yingjie Xiao, Ping Li, Dongyue Lin, Haotao Li, Qiang Fang, Shanyan Yu, Mingqiang Liu from the Shantou University for their contributions in the data collection. We also thank the participants for their time and efforts.

## Ethical issues

 The study was approved by the Survey and Behavioral Research Ethics Committee of the Chinese University of Hong Kong (No. SBRE-20-094).

## Competing interests

 Authors declare that they have no competing interests.

## Authors’ contributions

 Conceptualization: JTFL and YY; Methodology: YY, JTFL; Investigation: SL, SW, JZ, GZ, LLL, and LPL; Software: YY; Formal analysis: YY; Data curation: YY and SL; Validation: JTFL; Resources: JTFL; Writing-original draft: YY, JTFL, and PM; Writing-review and editing: YY, JTFL, and PM; Supervision: JTFL; Funding acquisition: JTFL.

## Funding

 The study was supported by internal research funding of the Centre for Health Behaviours Research. The funding source has no role in this study.

## Supplementary files

Supplementary file 1 contains Figure S1.
