# Supplementary material for: Prosociality and Social Responsibility Were Associated With Intention of COVID-19 Vaccination Among University Students in China
Source: Int J Health Policy Manag. 2021 Jun 26;11(8):1562–9. doi: 10.34172/ijhpm.2021.64 (PMC9808345; doi:10.34172/ijhpm.2021.64)
Supplement: Supplementary file 1 — contains Figure S1. [file ijhpm-11-1562-s001.pdf]

Article title: Prosociality and Social Responsibility Were Associated With Intention of COVID-19 Vaccination Among University Students in China

Journal name: International Journal of Health Policy and Management (IJHPM)

Authors' information: Yanqiu Yu [1], Sitong Luo [1], Phoenix Kit-han Mo [1], Suhua Wang [2], Junfeng Zhao [3], Guohua Zhang [4], Lijuan Li [5], Liping Li [6], Joseph Tak-fai Lau [1, *]

[1]Centre for Health Behaviours Research, JC School of Public Health and Primary Care, The Chinese University of Hong Kong, Hong Kong, China.

[2]Graduate School of Baotou Medical College, Baotou Medical College, Baotou, China.

[3]Department of Psychology, School of Education, Henan University, Kaifeng, China.

[4]Department of Psychology, School of Psychiatry, Wenzhou Medical University, Wenzhou, China.

[5]School of Public Health, Dali University, Dali, China.

[6]Shantou University Medical College, Shantou, China.

(*Corresponding author: jlau@cuhk.edu.hk)

Supplementary file 1.

Supplementary Figure of "Prosociality and Social Responsibility were Associated with Intention of COVID-19 Vaccination among University Students in China"

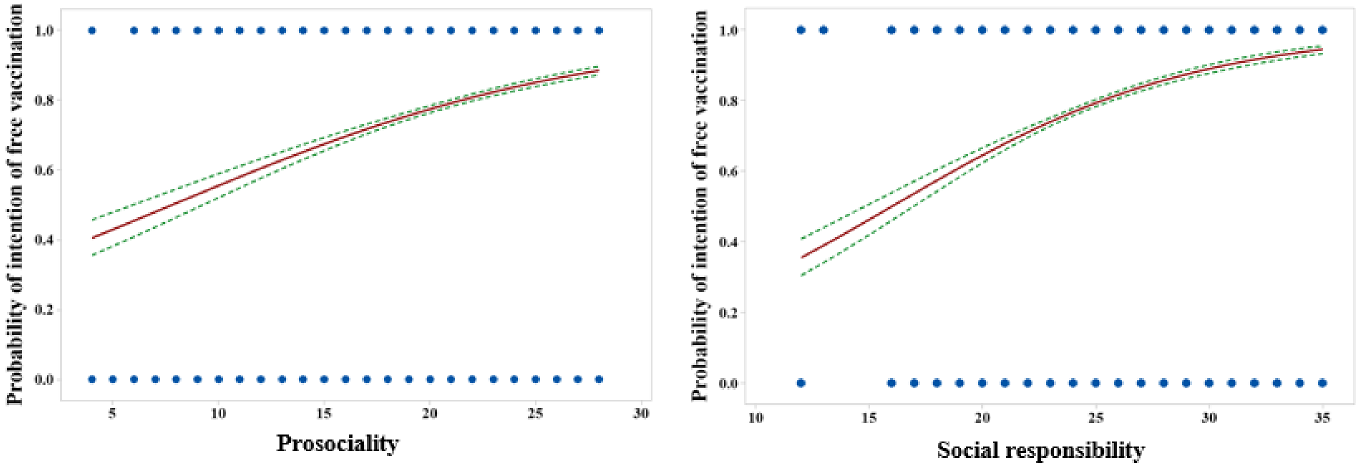

(a) Behavioral intention of free COVID-19 vaccination

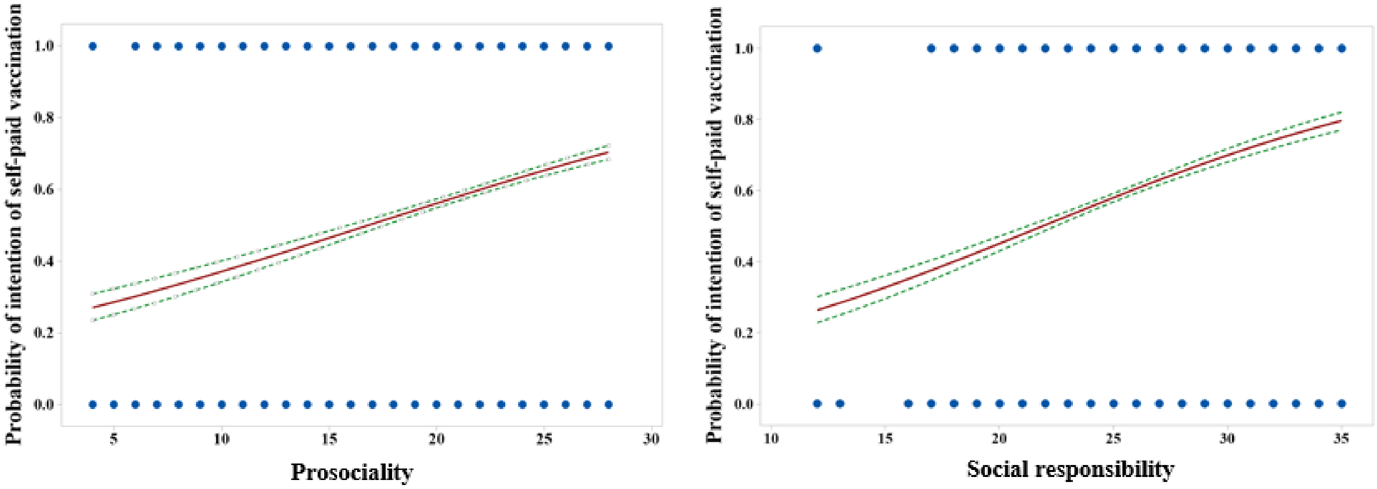

(b) Behavioral intention of self-paid COVID-19 vaccination

Figure S1. Logit fitting plots of the relationship between prosociality/social responsibility and behavioral intention of free/self-paid COVID-19 vaccination
